# Workloads of forward and backline adolescent rugby players: a pilot study

**DOI:** 10.17159/2078-516X/2020/v32i1a7427

**Published:** 2020-01-01

**Authors:** D Barnard, L Pote, C J Christie

**Affiliations:** Department of Human Kinetics and Ergonomics, Rhodes University, Grahamstown, South Africa

**Keywords:** rugby union, session rating of perceived exertion, workload monitoring, AC ratio, cost effectiveness

## Abstract

**Background:**

There is minimal research on workloads of adolescent rugby players. Therefore, the main aim of this study was to determine the workloads placed on a cohort of South African adolescent rugby players (n = 17), during an in-season period.

**Methods:**

Session RPE ratings were collected daily, 30 minutes after the training session concluded, during an 11-week in-season period. The training load was calculated as the session ratings of perceived exertion multiplied by the session’s duration (min).

**Results:**

The main finding of the study was that the adolescents in this investigation had similar workloads to elite players but higher workloads than other studies on adolescent rugby players. The forwards (3311±939 arbitrary units; AU) had a higher workload than backline players (2851±1080 AU). There was no difference between forwards and backline players with regards to the acute:chronic workload ratio.

**Conclusion:**

Workloads are high in these adolescent players, particularly in the forwards, and are similar to the workloads of elite level rugby players.

Rugby has become one of the fastest growing team sports in the world ^[1,2]^. As more players participate and the seasons get longer, more injuries are being reported ^[1,2]^. Injuries (excluding catastrophic injuries and concussion) have been linked to over- or undertraining ^[3]^. There is a paucity of literature on workloads placed on adolescent rugby players and most of the understanding comes from rugby league ^[4]^. A study on the workload of senior elite level players showed that forwards had a higher workload than backline players ^[5]^. In contrast, Phibbs et al. ^[6]^ found that in elite adolescent rugby players, backline players had higher workloads than forwards, which was similar in respective years ^[7,8]^ and lower than those placed on elite players ^[5]^. This is most likely due to the shorter duration of match play for elite adolescents in the United Kingdom which was reported to be a mean of 50 ± 44 minutes ^[6,7]^. This is in contrast to a match duration of 80 minutes at an elite level and within senior South African schoolboy rugby (70 minutes). It must be acknowledged that elite training durations are not publicly available. According to the authors’ knowledge there has also been minimal reported research on practice durations. Although the workloads on rugby players is high, priority has been given to the prevention of impact injuries rather than on managing workloads ^[8]^.

A combination of measuring internal and external loads is useful as they provide a holistic understanding of what stressors are being placed on the athlete ^[8]^. This holistic understanding optimises training and performance ^[8]^. Using the internal, subjective measure of session Rating of Perceived Exertion (sRPE) and the external, objective measure of session duration is a very cost-effective method used to monitor an athlete’s load. This is particularly useful in a school setting because these two variables do not require specialised equipment. Session RPE is typically measured using the 10-point Likert scale adapted by Forster et al ^[9]^. sRPE is both a valid and reliable measurement for monitoring the workloads in athletes ^[9,10]^ with an error rate of 4.3% in adolescents ^[10]^. Furthermore, it is only as accurate as the player’s understanding of the scale, so adequate explanation and habituation to the scale needs to be provided ^[10]^.

The use of sRPE and training duration to calculate training load also allows for the calculation of the acute:chronic ratio ^[3]^. This ratio allows the players’ workload to be monitored throughout a season. Training load can be both the acute and the chronic load placed on an athlete. Acute refers to one training session load or one week’s training load ^[3]^. Chronic refers to a longer period encompassing, for example, a two-week rolling average which is the cumulative load for two weeks of training, while a four-week rolling average is the cumulative of four weeks training ^[3]^. The usual chronic load is a period of four weeks; however, the two-week chronic load can be used as it has been used in the purpose of return-to-play protocols ^[3]^.

An optimal acute:chronic ratio is between 0.8 and 1.3 ^[3]^ which is referred to as the ‘sweet spot’ which is where injury risk is purported to be at its lowest ^[3,8,11]^. When the ratio is 1.5 or higher it is referred to as the ‘danger zone*’* which has a high injury risk ^[3,11]^. When the ratio is below 0.8 the athletes are at a point where they are undertraining, thus leading to a higher risk of injury ^[3]^.

Recent research has shown that there are conflicting views regarding the acute:chronic ratio and whether or not it is actually a good predictor for injury. A new method uses an exponentially weighted moving average, which places more emphasis on recent workload compared to the whole period’s workload ^[12]^. However, the rolling average method is more appropriate for a school setting as it considers the whole portion of load ^[3]^.

It is evident that workload monitoring may be an effective tool to monitor fatigue and injury risk in athletes ^[8]^. However, there are only a few studies which have attempted to measure workloads on adolescent rugby players and none, to theses authors’ knowledge, within a South African context. Therefore, the purpose of this study was to determine the workloads placed on adolescent rugby players in the in-season period. This serves as a pilot study to prepare for future studies on this topic.

## Methods

### Players

In this study a convenience sample from a private school in Grahamstown, South Africa was used. The sample included seventeen male, first team squad players between the ages of 16 to 19 years (stature: 1.74 m; mass: 77.4 kg). The study was approved by the Rhodes University Ethical Standards Committee for research involving human participants (1075008). Approval was obtained from the school and written informed consent was obtained from all players over the age of 18 years. Written consent was obtained from the parents of those players under the age of 18 years. Furthermore, players were also required to provide assent.

### Study design

The study was a prospective cohort study which measured match and training load (sRPE and session duration) daily, 30 minutes after the end of the training session, over eleven weeks during the in-season period. The in-season period was used for pragmatic reasons, as prior to this, players were on holiday or participating in other sports. Although adolescent rugby union players can train all year round, the data collection period in this study was selected to represent the normal South African school rugby competitive season. There was no minimum duration required for training or matches and all were included in the analyses.

### Collection period

In-season data were obtained daily during the 11-week period. During this period there was only one other sport that could be played, namely hockey. These workloads were included in the total weekly load for the relevant players.

### Workloads monitored

Session RPE and duration of the session where used respectively to calculate workload. The RPE scale used was the adapted version by Forster et al. ^[9]^. Participants were provided with a hard copy of the scale and were required to rate their effort for perceived exertion thirty minutes after the conclusion of the session. This delay was to ensure that the measurement reflected the whole session and not only the intensity of the last activity performed. The duration of the session was timed by the coach, from the moment the warmup started until the cool-down finished.

### Measurements and quantifications

Session RPE and session duration were used to calculate training or match load as follows ^[9]^:


Equation 1 $$\begin{array}{l} \text{Training Load }(\text{TL})=\text{Session Rating of}\\ \text{Perceived Effort }(\text{sRPE})\times \text{Duration of}\\ \text{session }(\text{Minutes}) \end{array}$$

The acute:chronic ratio was calculated by dividing the acute load by the chronic load. In this case the acute workload consisted of one week’s load and the chronic load referred to two weeks of accumulative load.


Equation 2 Acute:Chronic (a:c)=Acute load/Chronic load

The two-week rolling average was used to allow for sufficient data points for comparative purposes as the data collection period was only eleven weeks. However, it is noted that the four-week rolling average is the optimal average to use and is a limitation of the current study.

### Procedures

There was a four-week habituation period before the start of the study. The participants were habituated to the session RPE scale after every practice or activity completed. The rating took place thirty minutes after the practice or match. Time of session was manually recorded by the researcher. The sessions included all the sports players’ participation in and any other training they undertook, which varied from player to player. The focus of this period was to ensure the players understood ratings of perceived effort. Thereafter, for the 11-week in-season period, times of their daily match or training sessions and sRPE were recorded.

### Statistical analysis

All data were entered to a customised spreadsheet (Microsoft Excel, Microsoft, Redmond, USA). Daily data were summated to provide weekly player match and training loads, as well as to quantify week-by-week changes of acute:chronic workload ratios. Analyses were done in R 2.14. ^[13]^. The data were not normally distributed. A Kruskal-Wallis test and one-way ANOVA were used to compare forwards versus backline players. Cohen’s *d* effect sizes (ES) were used to establish the degree of difference between forwards and backline players, as well as between match and practice data. The criteria that were used for interpreting effect size was <0.2 trivial, 0.2–0.6 small, 0.6–1.2 moderate, 1.2–2.0 large, and > 2.0 very large ^[14]^. Significance was set at p<0.01.

## Results

Practice duration (86.05 ± 33.54 minutes) was longer than the match duration (75.09 ± 19.90 minutes), with an effect which was small (*ES*=0.35). This was despite the fact that sRPE was lower for practices (6.3 ± 1.7 AU) compared to matches (7.2 ± 1.6 AU), with a small effect (*ES*=0.53).

**Fig. 1:**
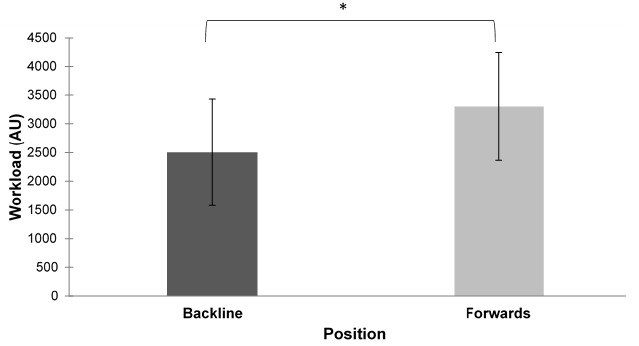
Mean ± standard deviation overall workloads of forwards and backline players over an 11-week period. * indicates significance (p<0.01).

**Fig. 2:**
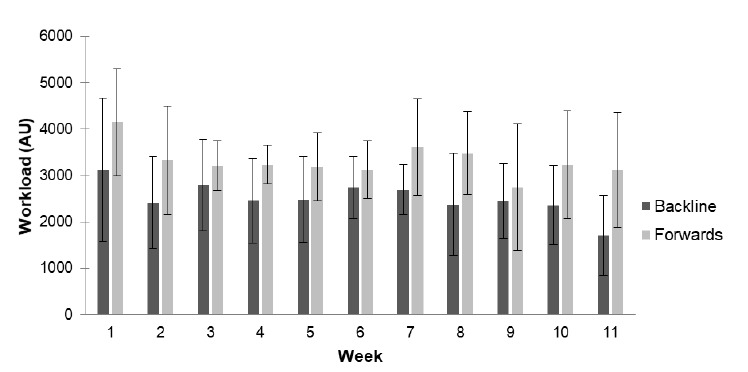
Weekly mean ± standard deviation workload (AU) differences between forward and backline players

**Fig. 3:**
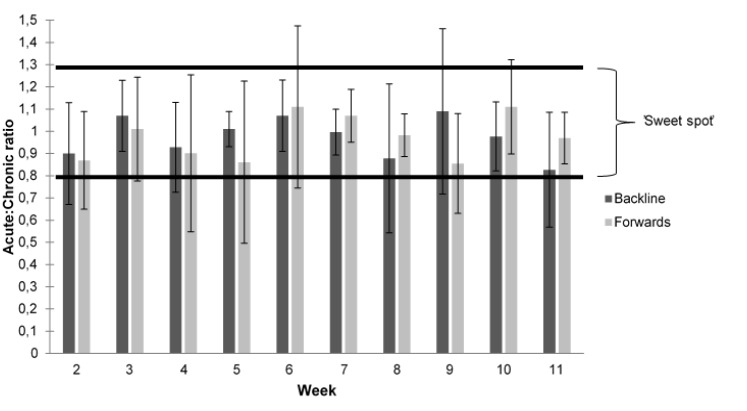
Mean ± standard deviation individual weekly acute:chronic ratio differences between forward and backline players.

Overall, workload for the 11-week period was significantly higher (p<0.01; *ES*=0.86) for the forwards compared to the backline players (Fig. 1). In contrast, there was no intra-week difference or differences between positions (Fig. 2). The mean workload for backs was 2507 ± 928 AU and 3311 ± 939 AU for the forwards. The mean workload for all the players was 2851 ± 1080 AU. Workloads varied weekly for both forwards and backs (Fig. 3).

**Fig. 4:**
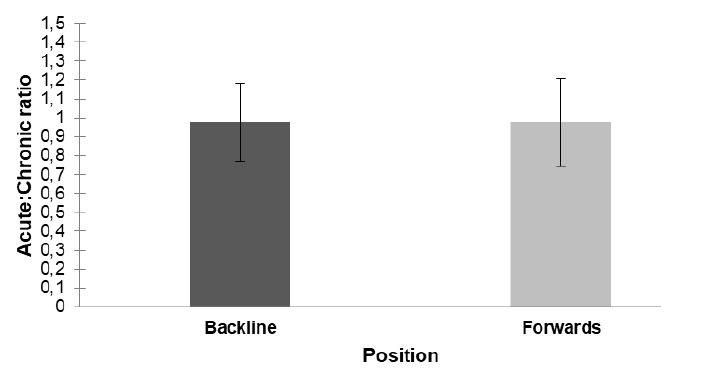
Mean ± standard deviation overall acute:chronic ratio between forwards and backline players over an 11-week period.

The mean acute:chronic ratio for the backs and forwards during the 11-week period was the same at 0.97 (*ES*=0.01) with a standard deviation of ± 0.21 for the backs and ± 0.23 for the forwards (Fig. 4). Week 1 was significantly (p<0.01) higher compared to all the other weeks and thereafter there was no difference (Fig. 4).

**Fig. 5:**
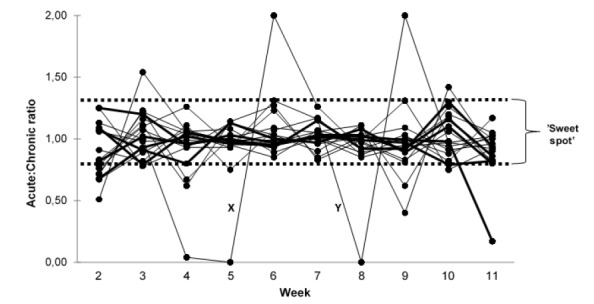
Total acute:chronic ratios for individual players over an 11-week period.

There was a total of 170 observations represented as acute:chronic ratios (Fig. 5). There were 20 observations (12%) that were not considered in the ‘sweet spot’ (0.8–1.3) ^[3]^ and there were only six observations (4%) that were above the ‘sweet spot’. Therefore, there were 144 observations (85%) in the recommended zone. There were only three players that remained in the ‘sweet spot’ throughout the 11-week period.

In summary the forwards had a significantly higher workload than backline players but acute:chronic ratios were similar.

## Discussion

The most important finding of this study was that workloads were similar to those placed on elite level players ^[5,6]^. Furthermore, and similar to other studies, forwards had higher overall workloads compared to backline players ^[5]^. Another important finding was that the mean workload of all players in this study (2851 ± 1080 AU) was higher than that of previous studies on adolescent players ^[6,7]^. It should be noted, that the players in the Phibbs et al.^[6]^ study, were classified as elite adolescent players who were part of an academy, whereas the players in this and the Phibbs et al.^[7]^ study were schoolboys. This is definitely an area that requires further investigation to determine whether rugby players at school level in South Africa have higher injury prevalence and whether this could be linked to workloads. Or whether countries like the United Kingdom could increase workloads to help the body better adapt. This is an interesting debate as it may also be linked to retention into senior level rugby. Higher workloads at school level may be a deterrent to continuation into higher levels of rugby.

The fact that forwards experience more load than backs is not unexpected as studies on adolescent ^[8]^ and adult players respectively ^[5]^ have found that forwards are involved in more high intensity activities. This has been directly assessed in elite adult forwards who were reported to have a higher energy expenditure than backline players ^[5]^. Forwards are also involved in more static bouts as a result experience higher workload ^[15]^. Anecdotally there is the perception that South African rugby favours a more forwards-based game, which relies on the size and power of forwards to drive a game compared to other countries. This may partly explain why forwards in this study had a greater load as this type of strategy is also favoured at the adolescent level. Interestingly, other studies at the non-adolescent level have also reported higher workloads in forwards compared to backs ^[5]^, which is in contrast to Phibbs et al.^[6]^ who reported higher workloads in backline players at the adolescent elite level. This is clearly something that needs to be investigated further. A possible reason for this contrast is that the rugby structures for adolescents in South Africa could be different to the structures in other countries. Also, the structures for non-elite adolescents may be different to those at an elite level.

When looking at overall workload in this study (2850 ± 1080 AU), it is higher than reported by Phibbs et al. ^[6,7]^ (1217 ± 367 AU and 1210 ± 571 AU respectively). The most likely reason for this is the differences in practice and, particularly, match duration. The players in this study had a longer mean match time (76 vs. 50 minutes) and similar practice time (172 vs. 178 minutes per week) compared to the players in the Phibbs et al. study ^[7]^. Why this is the case is not known but it is something that needs to be considered and investigated further, as this may be a way in which workloads can be optimised at the adolescent level to ensure that player injuries are minimised.

Most players were within the ‘sweet spot’ range which is in contrast to the players in the study of Phibbs et al.^[7]^. The findings show that a higher percentage (85%) of observations were in the zone compared to Phibbs et al.^[7]^ who had 60% observations in this range. Three players in this study remained continuously in the zone, which is in contrast to Phibbs et al. ^[7]^ who showed that no players were consistently in the zone. Compared to Phibbs et al. ^[7]^ the current study had few players both above (4% vs. 13%) and below (12% vs. 26%) the ‘sweet spot’. This suggests the current cohort was optimally managed in terms of workload.

It must be noted that there were two participants who had an injury during the 11-week period. The first participant (forward player) had a concussion during a match in week three which then prevented him training in the weeks thereafter. This player thus had very low acute:chronic ratios during weeks four and five respectively (Fig. 5). The second participant (backline player) was injured during a hockey match in week seven which prevented him from training during week eight, so he had an acute:chronic ratio of zero in that week (Fig. 5). With both players missing practices, it caused a decrease in acute:chronic ratios and these players fell below the ‘sweet spot’ (Fig. 5). As to be expected, they had a spike in their acute:chronic ratios during weeks six and nine respectively, which was their return-to- play week (Fig. 5).

## Conclusion

In conclusion, this study found that most players were training within the recommended range ^[3]^. However, their workloads were as high as elite level players and particularly high in the forwards. This is something that needs to be monitored and managed by both coaches and medical teams.
